# CYP46A1‐Targeted Treatment Alleviates Long‐Term White Matter Injury Following Traumatic Brain Injury by Promoting Cholesterol Metabolic Clearance and Remyelination

**DOI:** 10.1002/cns.70984

**Published:** 2026-06-15

**Authors:** Lin Li, You Shi, Qing Luo, Peiwen Guo, Taotao Jin, Xufang Ru, Zhouyang Jiang, Yin Niu, Wenyan Li, Yujie Chen, Zhi Chen

**Affiliations:** ^1^ Department of Neurosurgery Southwest Hospital, Third Military Medical University (Army Medical University) Chongqing China; ^2^ Department of Neuro‐Oncology Chongqing University Cancer Hospital Chongqing China; ^3^ Department of Ultrasound Chonggang General Hospital, Chongqing University of Posts and Telecommunications Chongqing China; ^4^ Chongqing Key Laboratory of Intelligent Diagnosis, Treatment and Rehabilitation of Central Nervous System Injuries, Southwest Hospital, Third Military Medical University (Army Medical University) Chongqing China; ^5^ Chongqing Clinical Research Center for Neurosurgery, Southwest Hospital, Third Military Medical University (Army Medical University) Chongqing China

**Keywords:** cholesterol metabolism, CYP46A1, liver X receptor, traumatic brain injury, White matter injury

## Abstract

**Aim:**

Cholesterol plays a critical role in repairing white matter injury (WMI) following traumatic brain injury (TBI). The enzyme cholesterol 24‐hydroxylase (CYP46A1) regulates the removal of cholesterol by converting it into 24(S)‐hydroxycholesterol (24OHC). Although CYP46A1 has neuroprotective effects on various central nervous system disorders, its effect on WMI remains unclear.

**Methods:**

Adult male C57BL/6 mice underwent controlled cortical impact to model TBI. Experiments using the CYP46A1 activator efavirenz and CYP46A1^−/−^ mice were utilized to elucidate the function of CYP46A1. Neurological function, WMI, and cholesterol metabolism were evaluated, and the mechanisms through which CYP46A1 affects these processes were explored.

**Results:**

Efavirenz markedly improved outcomes and preserved white matter structure after TBI by increasing microglial phagocytic activity and myelin debris clearance, along with promoting oligodendrocyte precursor cell remyelination. Furthermore, efavirenz promoted cholesterol export by increasing 24OHC levels and activating liver X receptors (LXR). However, these neuroprotective effects of Efavirenz were partially diminished when CYP46A1 was knocked down or when LXR activity was blocked.

**Conclusion:**

Efavirenz administration promotes functional neurological recovery and sustains white matter integrity in TBI through the regulation of cholesterol homeostasis and the promotion of remyelination processes.

## Graphical abstract

Our findings demonstrate that CYP46A1 plays a critical role in regulating cholesterol homeostasis in microglia and that the targeting of CYP46A1 may offer a viable therapeutic approach for traumatic brain injury (TBI). The CYP46A1 activator Efavirenz reduces white matter damage by increasing the levels of the cholesterol metabolite 24OHC, thereby stabilizing the cholesterol balance after TBI. Furthermore, Efavirenz could serve as a promising treatment for TBI by restoring 24OHC/LXR signaling.

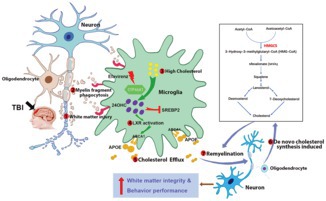

**Abbreviations**
24OHC: 24‐hydroxycholesterol
ABCA1: ATP‐binding cassette transporter A1
ABCG1: ATP‐binding cassette transporter A1
ApoE: Apolipoprotein E
BrdU: Bromodeoxyuridine
CC: corpus callosum
CCI: controlled cortical impact
DTI: diffusion tensor imaging
EC: external capsule
FA: fractional anisotropy
HMGCS1: HMG‐CoA synthetase
LAMP1: lysosomal‐associated membrane protein 1
LC–MS: liquid chromatography–mass spectrometry
LDL: Low‐Density Lipoprotein
LXR: liver X receptors
MBP: myelin basic protein
MEPs: motor evoked potentials
NOR: the nodes of Ranvier
OPC: oligodendrocyte precursor cells
PPAR: Peroxisome Proliferator‐Activated Receptor
SREBP2: Sterol Regulatory Element‐Binding Protein 2
TBI: Traumatic brain injury
TEM: transmission electron microscopy
WMI: white matter injury

## 1 Introduction

Traumatic brain injury (TBI) poses significant global healthcare, societal, and economic burdens because of its high fatality rates, associated complications, and persistent functional impairments [1, 2]. Among the various pathological injuries caused by impact (such as intracerebral hemorrhage, necrotic‐ischemic injury, and tissue avulsion), white matter injury (WMI) is caused by direct mechanical damage to axons either multifocally or secondary to brain injury [3]. Currently, there are no approved pharmacological treatments specifically targeting white matter damage in TBI patients.

The myelin sheath has a high lipid content, including elevated concentrations of cholesterol and glycosphingolipids. Demyelination represents a key pathological feature of TBI, with persistent demyelination and the production of fragments of myelin debris being observed [4]. Within the central nervous system, microglia serve as the main phagocytic cells and play an essential role in clearing cellular debris from injured regions. In demyelinating CNS disorders such as multiple sclerosis (MS), microglia function as the principal cells that are responsible for eliminating myelin breakdown products [5].

Oxysterols are oxidized forms of cholesterol that play a role in modulating cholesterol homeostasis. These compounds can be obtained from dietary sources or synthesized endogenously through cholesterol metabolism. The major classes of oxysterols include 24‐hydroxycholesterol (24OHC), 25‐hydroxycholesterol (25OHC), 27‐hydroxycholesterol (27OHC), and cyclic oxysterols [6]. In addition to their involvement in cholesterol regulation, oxysterols influence various signaling cascades, such as the Hedgehog, Wnt, and MAPK pathways. In neurodegenerative conditions, oxysterols engage with specific molecular targets, particularly liver X receptors (LXRs) [7], to exert their biological effects. Research has demonstrated that 27OHC can inhibit prostate cancer progression by reducing intracellular cholesterol concentrations [8]. Similarly, disrupted cholesterol balance has been linked to unfavorable outcomes in certain liver cancer subtypes [9]. In TBI, the maintenance of the microglial cholesterol balance is critical for myelin repair, as evidenced by two key findings. First, microglia clear myelin fragments to create a favorable environment for regeneration [5]. Second, endogenous oxysterols increase cholesterol export [10]. However, the exact mechanisms underlying cholesterol dysregulation in TBI‐associated microglia (particularly concerning oxysterols) remain unclear.

Cholesterol metabolism is tightly regulated by a network of proteins that primarily involve two key transcription factors: liver X receptors (LXRs) and sterol regulatory element‐binding proteins (SREBRs) in mammals. These factors play crucial roles in various cholesterol‐related processes, including uptake, efflux, biosynthesis, and esterification [11]. When excessive cellular cholesterol levels are present, LXRs are activated, thus promoting the expression of ABCA1 and ABCG1, which facilitates cholesterol removal and the degradation of LDL receptors [12]. Due to their central role in cholesterol homeostasis, these pathways have emerged as promising therapeutic targets for reducing white matter injury in TBI models [13].

TBI triggers the accumulation of excess cellular cholesterol because of the clearance of myelin debris, thus suggesting that the modulation of cholesterol metabolism could offer a potential therapeutic approach. Previous research has demonstrated that CYP46A1 expression significantly increases by day 7 postinjury, with microglia exhibiting an 84% increase in Cyp46 levels [14]. CYP46A1 facilitates cholesterol elimination through its conversion into 24OHC [8]. In this study, we investigated whether enhancement of the activity of CYP46A1 could improve TBI outcomes. Our findings reveal that the pharmacological activation of CYP46A1 using Efavirenz (an FDA‐approved anti‐retroviral medication) promotes neurological recovery and preserves white matter integrity, which is likely achieved through the 24OHC/LXR pathway. These results highlight the critical role of CYP46A1 in regulating cholesterol balance and suggest that the targeting of this axis may represent a promising strategy for TBI treatment.

## 2 Materials and Methods

### 2.1 Experimental Animals

All the animal experiments were reviewed and approved by the Laboratory Animal Welfare and Ethics Committee of the Army Medical University (AMUWEC20224973); moreover, the experiments were performed according to the Guidelines for the Care and Use of Laboratory Animals of the National Institutes of Health, and results were reported in accordance with the ARRIVE 2.0 guidelines. The mice were housed 5 per cage in a temperature‐, humidity‐, and light/dark‐controlled environment; additionally, food and water were always available.

Adult male C57BL/6 wild‐type (WT) mice (20–25 g) were purchased from the Experimental Animal Center of Army Medical University. Adult male CYP46A1 KO mice (20–25 g) were purchased from Jackson Lab (B6; 129S7‐Cyp46a1tm1Rus/J, Stock No. 017759). The animals were anesthetized with isoflurane (3% induction, 2% maintenance) before all of the experiments were performed. Brain samples were harvested after the animals were euthanized with pentobarbital sodium (0.3%, 40 mg/kg).

Detailed experimental methods are provided in the Supplemental Methods.

### 2.2 Statistical Analysis

The results are presented as the mean ± standard deviations. Normality was assessed by using the Shapiro–Wilk test. Group comparisons were performed with one‐way ANOVA; moreover, the Tukey's post hoc test was applied for multiple comparisons. For repeated measurements, a two‐way repeated‐measures ANOVA with the Tukey's post hoc test was used. All of the statistical analyses were performed by using GraphPad Prism (v9.1.0, USA), with the significance threshold set at *p* < 0.05.

## 3 Results

### 3.1 CYP46A1 Activation Improves Neurological Function and Promotes Brain Electrophysiological Recovery After TBI

**FIGURE 1:**
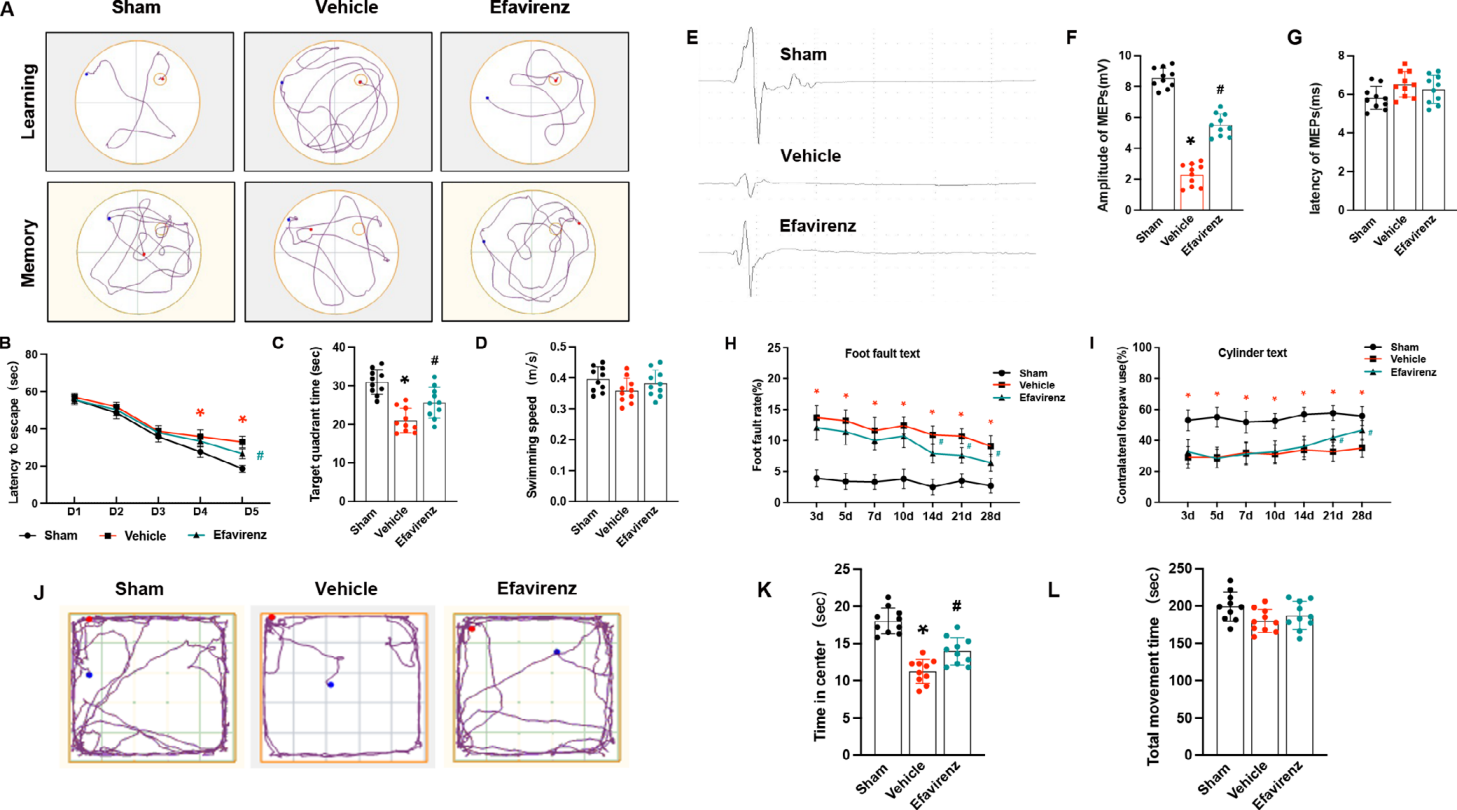
CYP46A1 activation promoted neurological recovery after TBI. (A–D) Efavirenz alleviated learning and memory impairments, as evaluated via the Morris water maze test at 28 days after TBI; *n* = 10/group. (E–G) MEPs indicated that Efavirenz affected MEP amplitudes (F) but not MEP latencies (G, *p* > 0.05); *n* = 10/group. (H, I) The results of the foot fault test and cylinder test were calculated; *n* = 10/group. (J–L) Open field test results suggested that Efavirenz mitigated anxiety‐like behaviors at 28 days after TBI; *n* = 10/group. *p < 0.05 versus the sham group; *#p* < 0.05 versus the Vehicle group.

Cognitive function was evaluated by using the Morris water maze test between 23 and 28 days after TBI. During the training phase, both Vehicle‐ and Efavirenz‐treated animals exhibited significantly longer escape latencies compared with sham control animals on days 26 and 27 (Figure 1A,B, *p* < 0.05). However, compared with the Vehicle group, the Efavirenz group exhibited shorter platform‐finding times by day 27 (Figure 1A,B, *p* < 0.05). In the probe trial, compared with the Vehicle treatment, Efavirenz treatment significantly increased the preference for the target quadrant, indicating enhanced spatial memory retention (Figure 1A–C, *p* < 0.05). No significant differences in swimming velocity were observed among the groups, thereby confirming that motor performance did not affect the memory assessment results (Figure 1D, *p* > 0.05).

To assess the effect of CYP46A1 activation on motor function, researchers recorded motor‐evoked potentials (MEPs) at 28 days after TBI and analyzed both amplitude and latency parameters. Compared with the sham‐operated controls, the Vehicle‐treated animals exhibited significantly lower MEP amplitudes (Figure 1E,F, *p* < 0.05). Conversely, compared with Vehicle treatment, Efavirenz treatment resulted in higher amplitude values (Figure 1E,F, *p* < 0.05). No statistically significant differences were observed in MEP latency measurements among the experimental groups (Figure 1E,G, *p* > 0.05).

Neurological function is a critical indicator for assessing the prognosis of TBI. To investigate the therapeutic potential of Efavirenz in TBI, sensorimotor performance was evaluated by using foot fault and cylinder tests over a 28‐day period postinjury (Figure 1H,I). The results demonstrated that TBI caused significant sensorimotor impairments, as evidenced by elevated foot fault frequencies in the forelimbs (Figure 1H, *p* < 0.05). Notably, Efavirenz administration reduced these deficits, with improvement in forepaw accuracy becoming apparent 14 days after TBI (Figure 1H, *p* < 0.05). Additionally, TBI led to pronounced forelimb asymmetry, as reflected by the decreased use of the contralateral forepaw in the cylinder test (Figure 1I, *p* < 0.05). This imbalance was effectively mitigated by Efavirenz treatment, with significant recovery being observed from day 21 onward (Figure 1I, *p* < 0.05).

During the open field test, no significant differences were observed in total movement time across the groups during the experimental phase (Figure 1J,L, *p* > 0.05). However, compared with the sham group, the Vehicle‐treated group spent less time in the center (Figure 1J,K, *p* < 0.05). Notably, compared with Vehicle‐treated mice, mice treated with Efavirenz demonstrated increased central zone occupancy (Figure 1J,K, *p* < 0.05), thus suggesting that CYP46A1 activation mitigates anxiety‐like behaviors after TBI.

### 3.2 CYP46A1 Activation Promotes White Matter Integrity Preservation After TBI

White matter damage is a critical feature of TBI. To assess the structural changes in the paranodal and nodal regions observed at 28 days after TBI, we examined the effects of Efavirenz on white matter integrity using immunofluorescence. Double labeling for Caspr1 and Nav1.6 was performed to visualize the nodes of Ranvier (NOR) in the external capsule (EC) and corpus callosum (CC). Our findings revealed a significant reduction in NOR numbers in the ipsilateral EC (Figure S1A,B, *p* < 0.05) and CC (Figure S1D,E, *p* < 0.05) in the TBI group compared with those in the sham group. In contrast, compared with Vehicle treatment, Efavirenz treatment restored NOR density in these regions (Figure S1A,B,D,E, *p* < 0.05). Additionally, compared with that in the sham control group, the paranodal length in both the EC and the CC was notably reduced, and this shortening of paranodes was ameliorated by Efavirenz treatment (Figure S1A,C,D,F, *p* < 0.05).

**FIGURE 2:**
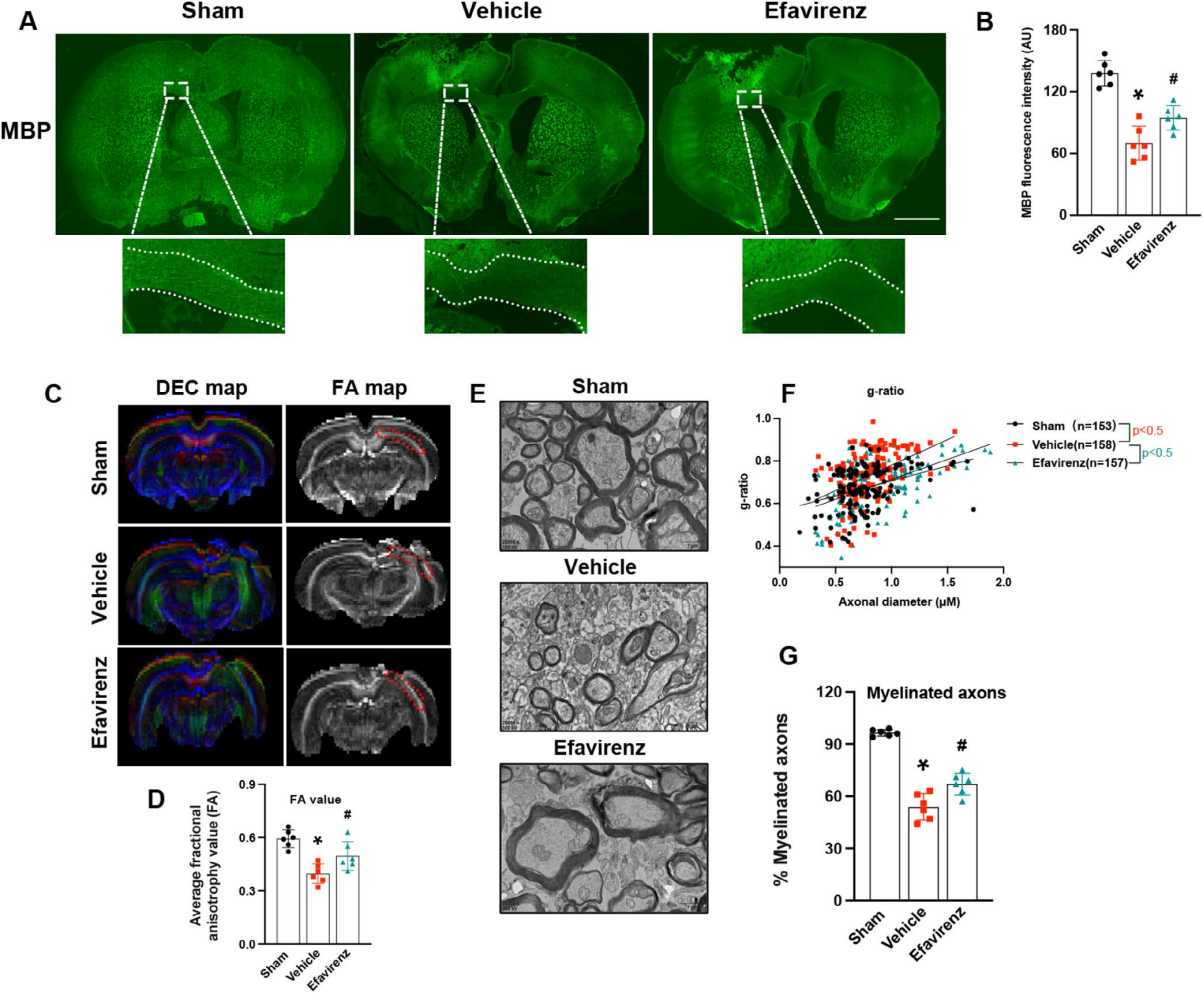
CYP46A1 activation alleviated white matter injury after TBI. (A, B) Efavirenz increased the MBP fluorescence intensity at 28 days after TBI; scale bar = 4 μm; *n* = 6/group. (C) Representative images showing DEC maps and FA maps around the lesion at 28 days after TBI; *n* = 6/group. (D) Quantification of the FA values around the lesion (red dashed box); *n* = 6/group. (E) The CC/EC area of each mouse was harvested for TEM analysis at 28 days after TBI; *n* = 6/group. (F) The g ratio was measured in the myelin of the mice; *n* = 6/group. (G) The proportion of myelinated axons was calculated; *n* = 6/group. *p < 0.05 versus the sham group; *#p* < 0.05 versus the Vehicle group.

To examine white matter integrity, MBP immunofluorescence staining was performed (Figure 2A,B). The findings revealed substantial white matter injury in the external capsule (EC)/corpus callosum (CC) regions adjacent to the CCI site after TBI, as evidenced by markedly reduced MBP expression compared with that in the sham group (Figure 2A,B, *p* < 0.05). Notably, compared with Vehicle treatment, Efavirenz treatment notably increased MBP immunoreactivity in this region (Figure 2A,B, *p* < 0.05).

To further investigate white matter microstructure alterations, DTI analysis was conducted. The FA values (which are indicative of white matter integrity) were significantly lower in the TBI group than in the sham control group (Figure 2C,D, *p* < 0.05). However, compared with Vehicle treatment, Efavirenz treatment led to a notable increase in FA values (Figure 2C,D, *p* < 0.05).

To assess the extent of myelin and axonal damage following TBI, transmission electron microscopy (TEM) was used to examine the CC/EC area. In the Vehicle group, significant myelin abnormalities (such as swollen and ballooned myelin sheaths, axonal degeneration, and a reduction in myelinated axons) were observed compared with those in the sham group. In contrast, these pathological alterations were mitigated in the Efavirenz‐treated group (Figure 2E). Further quantitative TEM analysis revealed that compared with the Sham group, the Vehicle group exhibited significantly reduced myelin thickness, as evidenced by elevated g ratios and a lower proportion of myelinated axons (Figure 2E–G, *p* < 0.05). Conversely, Efavirenz administration led to a reduction in the g ratio and an increase in the proportion of myelinated axons (Figure 2E–G, *p* < 0.05). These findings suggest that CYP46A1 activation plays a protective role against myelin degradation and axonal loss after TBI.

### 3.3 CYP46A1 Activation Inhibits Oligodendrocyte Apoptosis and Promotes Oligodendrocyte and Oligodendrocyte Precursor Cell (OPC) Proliferation After TBI

**FIGURE 3:**
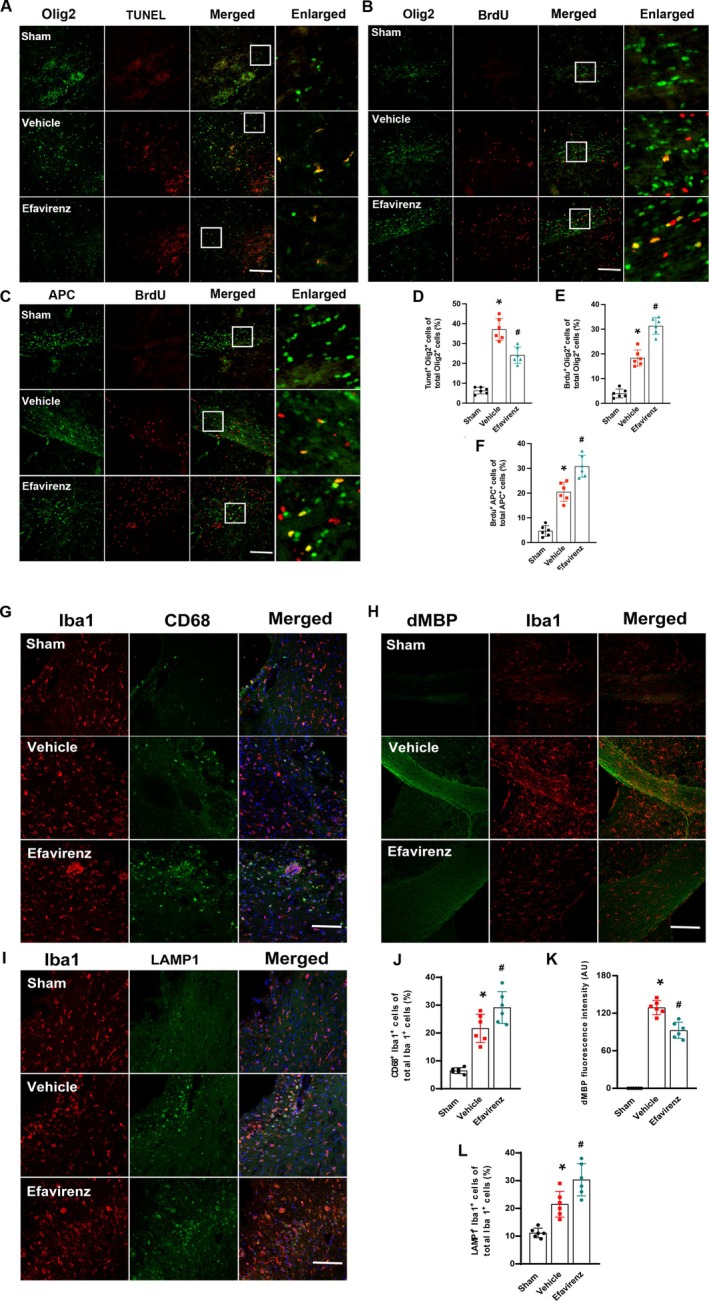
Efavirenz promoted OPC proliferation and microglial phagocytosis after TBI. (A–D) Colocalization of oligodendrocytes (Olig2, green) with TUNEL staining (red) and the number of Olig2^+^TUNEL^+^ cells in the external capsule on the injured side at 28 days after TBI; scale bar = 20 μm; *n* = 6/group. (B–E) Colocalization of oligodendrocytes (Olig2, green) with BrdU staining (red) and the number of Olig2^+^BrdU^+^ cells in the external capsule on the injured side at 28 days after TBI; scale bar = 20 μm; *n* = 6/group. (C–F) Colocalization of OPCs (APC, green) with BrdU staining (red) and the number of APC^+^BrdU^+^ cells in the external capsule on the injured side at 28 days after TBI; scale bar = 20 μm; *n* = 6/group. *p < 0.05 versus the sham group; *#p* < 0.05 versus the Vehicle group. (G–J) The colocalization of microglia (Iba1, red) with CD68 (green) and the number of CD68^+^Iba1^+^ cells around the lesion at 28 days after TBI; scale bar = 20 μm; *n* = 6/group. (H–K) The colocalization of Iba1 (red) with dMBP (green) and the dMBP immunofluorescence density in the external capsule on the injured side at 28 days after TBI; scale bar = 20 μm; *n* = 6/group. (I–L) Colocalization of Iba1 (green) with LAMP1 (red) and LAMP1^+^Iba1^+^ cells around the lesion at 28 days after TBI; scale bar = 20 μm; *n* = 6/group. *p < 0.05 versus the sham group; *#p* < 0.05 versus the Vehicle group.

CYP46A1 activation mitigated white matter damage after surgery, as evidenced by reduced myelin breakdown following TBI. Oligodendrocytes play a crucial role in the formation of the myelin sheath by enveloping axons, and this structure is essential for efficient bioelectrical signal transmission and maintaining neuronal function. Additionally, oligodendrocyte precursor cells (OPCs) mature into oligodendrocytes in the CNS [15]. In this study, we noted a greater number of apoptotic oligodendrocytes in the Vehicle group than in the sham group at 28 days after surgery. However, compared with Vehicle treatment, Efavirenz treatment significantly inhibited oligodendrocyte apoptosis (Figure 3A–D, *p* < 0.05).

To determine the effect of early oligodendrocyte loss on white matter recovery, we evaluated oligodendrocyte and OPC proliferation by analyzing Bromodeoxyuridine (BrdU) uptake in newly formed cells. Our findings revealed a marked increase in Olig2^+^BrdU^+^ and NG2^+^BrdU^++^ cells in the Vehicle group compared with those in the sham group (Figure 3B–F, *p* < 0.05). Furthermore, compared with Vehicle treatment, Efavirenz treatment led to a greater number of Olig2^+^BrdU^+^ and NG2^+^BrdU^+^ cells at 28 days after TBI (Figure 3B–F, *p* < 0.05).

### 3.4 CYP46A1 Activation Promotes Microglial Phagocytosis and Myelin Debris Clearance After TBI

Previous studies have demonstrated that microglial phagocytosis plays a crucial role in myelin regeneration across various central nervous system disorders. To assess microglial activation near the injury site, we performed dual labeling for Iba1 and CD68, with the latter marker being recognized as a marker of both lysosomal activity and microglial activation [16]. Compared with that in the sham group, the number of Iba1^+^CD68^+^ cells in the Efavirenz‐treated group was greater (Figure 3G–J, *p* < 0.05). These findings suggest that CYP46A1 activation increases microglial phagocytic capacity after traumatic brain injury.

Previous investigations have reported increased expression of lysosomal‐associated membrane protein 1 (LAMP1) in glial cells during neurological conditions such as Alzheimer's disease, thus underscoring its importance in phagocytic processes [17]. We evaluated microglial phagocytic activity through costaining for Iba1 and LAMP1. Compared with those in the sham group, the numbers of Iba1^+^LAMP1^+^ cells in the Efavirenz group were greater (Figure 3I–L, *p* < 0.05). Furthermore, dMBP immunofluorescence revealed more extensive myelin debris deposition in the Vehicle group than in the Efavirenz‐treated group (Figure 3H–K, *p* < 0.05). These findings suggest that CYP46A1 activation promotes microglial phagocytosis and the removal of myelin debris following TBI.

### 3.5 CYP46A1 Activation Reduces Microglial Cholesterol Accumulation by Regulating Microglial Sterol Metabolism‐Related Pathways After TBI

**FIGURE 4:**
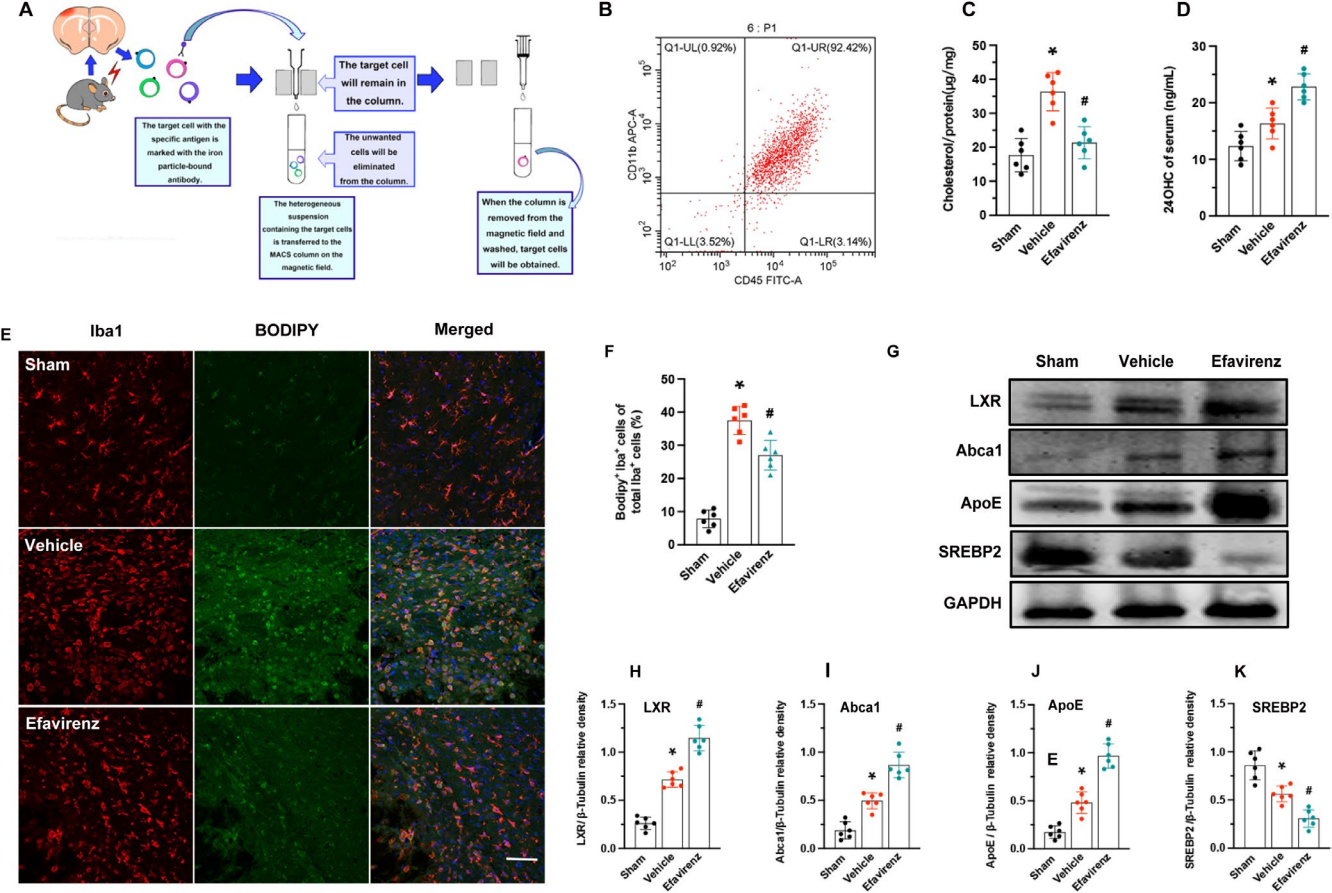
CYP46A1 activation reduced microglial cholesterol accumulation by regulating the 24OHC/LXR pathway after TBI. (A, B) Schematic diagram of the microglial sorting process and purity. (C, D) Cholesterol concentration of the sorted microglia and serum 24OHC concentration at 28 days after TBI; *n* = 6/group. (E, F) The colocalization of microglia (Iba1, red) with BODIPY (green) and the number of Iba1^+^BODIPY^+^ cells around the lesion at 28 days after TBI; scale bar = 20 μm; *n* = 6/group. (G–K) Western blot analysis of LXR, Abca1, ApoE and SREBP2 expression at 28 days after TBI. Protein samples were extracted from around the lesion; *n* = 6/group. *p < 0.05 versus the sham group; *#p* < 0.05 versus the Vehicle group.

Previous experiments and findings have indicated that the activation of CYP46A1 improves microglial phagocytic function and promotes oligodendrocyte proliferation, thereby reducing white matter damage. However, the exact mechanism of this effect remains unclear. CYP46A1 encodes sterol 24‐hydroxylase, which is an enzyme that converts cholesterol into 24‐hydroxycholesterol. Studies have demonstrated elevated Cyp46 levels seven days after traumatic brain injury, with microglia exhibiting an 84% increase in Cyp46 expression [14]. To investigate the metabolic changes in cholesterol and 24OHC in microglia, we isolated these cells near the injury site by using magnetic sorting at seven days after TBI (Figure 4A). Flow cytometry confirmed that the microglial purity was approximately 92% (Figure 4B). Our data revealed higher intracellular cholesterol levels in the Vehicle group than in the sham group, whereas Efavirenz treatment reduced cholesterol accumulation in the sorted microglia (Figure 4C, *p* < 0.05). Furthermore, serum 24OHC levels were elevated in the Vehicle group compared with those in the sham group but were significantly lower than those in the Efavirenz‐treated group (Figure 4D, *p* < 0.05).

Immunofluorescence analysis revealed that the number of BODIPY^+^Iba1^+^ cells with cholesterol accumulation was greater in the TBI group than in the sham group (Figure 4E,F). Interestingly, compared with the Vehicle‐treated group, the number of BODIPY^+^Iba1^+^ cells was reduced in the Efavirenz‐treated group (Figure 4E,F, *p* < 0.05). These findings aligned with the elevated CYP46A1 expression in microglia after TBI, wherein CYP46A1 activation significantly increased 24OHC levels in the serum. Together, these data suggest that Efavirenz promotes the metabolism of cholesterol into 24‐hydroxycholesterol, thereby facilitating its release into the peripheral circulation.

24OHC (a cholesterol metabolite) serves as an endogenous activator of liver X receptors (LXRs), thereby aiding in the regulation of cellular cholesterol homeostasis [12, 13, 14, 15, 16, 17, 18]. The LXR pathway in microglia is involved in lipid transport; moreover, it reduces neuroinflammation, inhibits cholesterol production, and supports remyelination following the occurrence of demyelinating disorders [5]. In this study, we observed higher expression levels of LXR and its downstream targets Abca1 and ApoE in the Vehicle group than in the sham group. However, these levels were further increased in the Efavirenz‐treated group. Conversely, Efavirenz treatment led to reduced protein expression of sterol regulatory element‐binding protein 2 (SREBP2) (Figure 4G–K, *p* < 0.05). These findings suggest that CYP46A1 activation through the LXR pathway promotes cholesterol efflux in microglia after TBI while simultaneously suppressing cholesterol synthesis through the downregulation of SREBP2.

### 3.6 CYP46A1 Depletion Abolishes the Effects of Efavirenz on Neurological Function, Sterol Metabolism‐Related Pathways and WMI After TBI

**FIGURE 5:**
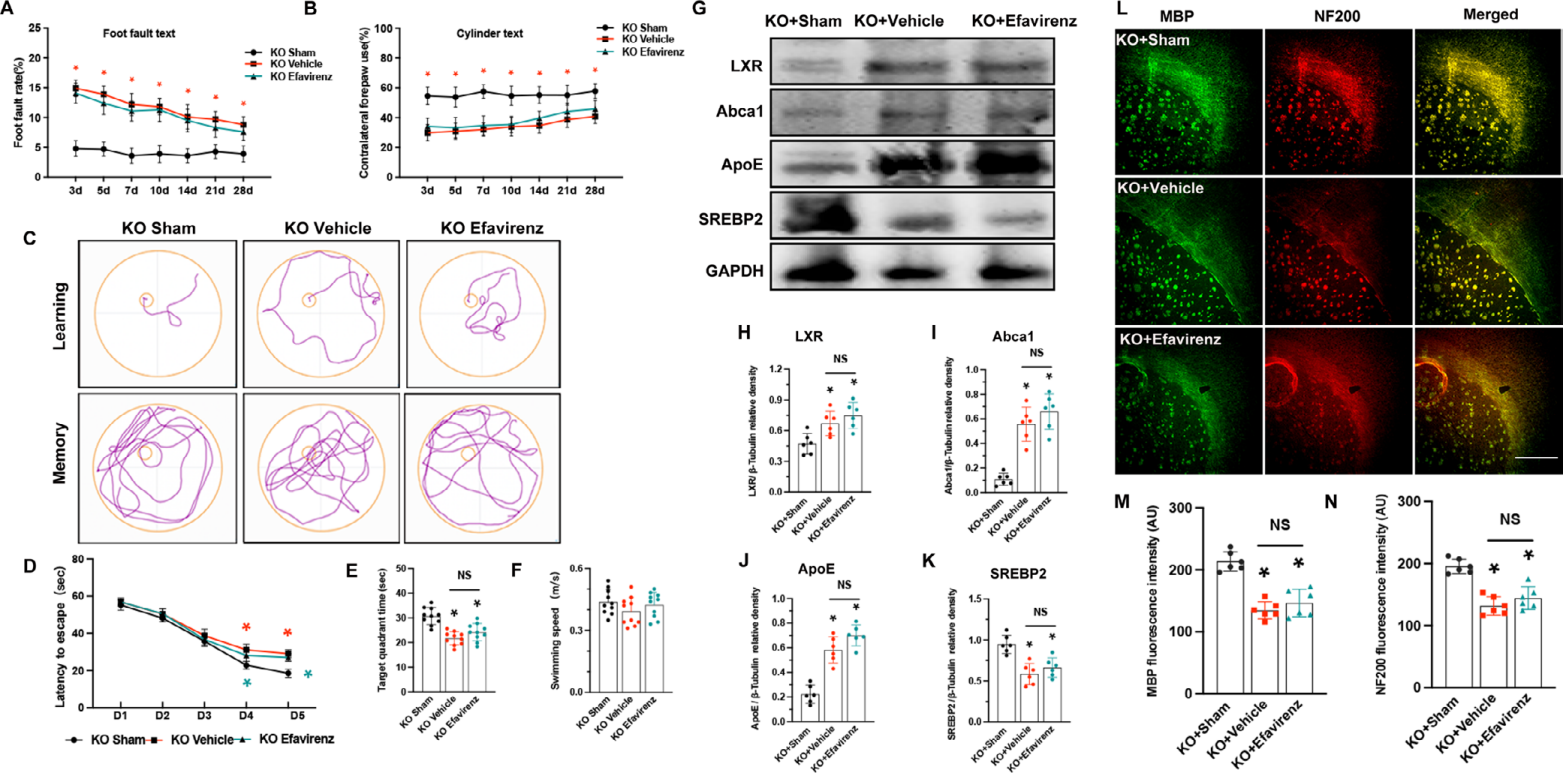
The neurological benefits of Efavirenz, along with its effects on sterol metabolism and white matter integrity after TBI, were eliminated upon CYP46A1 knockdown. (A, B) Foot fault tests and cylinder tests were performed; *n* = 10/group. (C–F) Assessment of cognitive deficits via the Morris water maze test between 23 and 28 days after TBI; *n* = 10/group. (L) Double immunostaining of MBP and NF200 in the EC at 28 days after TBI; scale bar = 20 μm; *n* = 6/group. (M, N) The fluorescence intensity of MBP and NF200; *n* = 6/group. *p < 0.05 versus the sham group; *#p* < 0.05 versus the Vehicle group.

To further explore the effects of CYP46A1 deficiency on the neuroprotective effects of Efavirenz, CYP46A1 knockout mice were divided into experimental groups. Efavirenz administration did not significantly affect performance in the foot fault or cylinder tests at various time points (3, 5, 7, 10, 14, 21, and 28 days) after TBI between the KO + Efavirenz and KO + Vehicle groups (Figure 5A,B). In the Morris water maze assessment of spatial learning, compared with the sham group, both the KO + Vehicle and KO + Efavirenz groups demonstrated longer times in finding the hidden platform on days 26 and 27 (Figure 5C,D, *p* < 0.05). However, compared with no treatment, Efavirenz treatment did not significantly alter the escape latency of CYP46A1‐KO mice on day 27 (Figure 5C,D, *p* > 0.05). Similarly, in the memory retention test, TBI reduced the time spent in the target quadrant; however, compared with the KO + Vehicle treatment, Efavirenz treatment did not improve this outcome (Figure 5C,E, *p* > 0.05). Additionally, no significant differences in swimming speed were observed across the three groups (Figure 5C–F, *p* > 0.05).

We observed higher expression levels of LXR, Abca1, and ApoE (but lower expression levels of SREBP2) in the KO + Vehicle group than in the KO + sham group. However, compared with the KO + Vehicle treatment, the Efavirenz treatment failed to increase the expression levels of LXR, Abca1, or ApoE and decrease the expression level of SREBP2 in the KO + Efavirenz group (Figure 5G–I, *p* > 0.05).

At 28 days after TBI, immunofluorescence staining for MBP and NF200 revealed significant white matter damage in the external capsule region. However, the damage observed in the KO + Efavirenz group was not mitigated in the KO + Vehicle group (Figure 5L–N, *p* > 0.05).

### 3.7 An LXR Inhibitor Impairs Microglia Cholesterol Efflux and Reduces White Matter Integrity via CYP46A1 Activation After TBI

**FIGURE 6:**
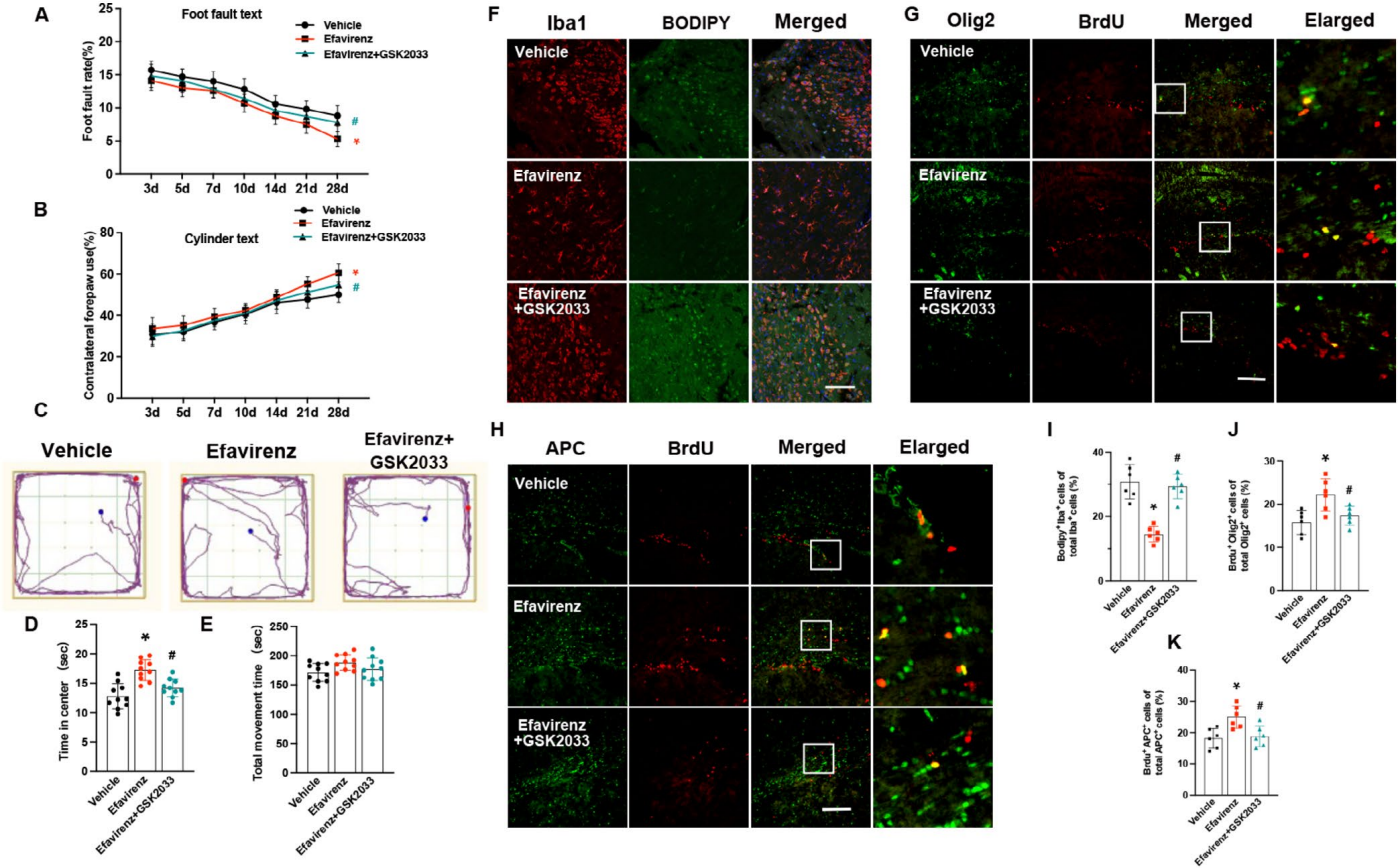
LXR inhibition impaired microglial cholesterol efflux and white matter integrity induced by Efavirenz after TBI. (A, B) Foot fault tests and cylinder tests were performed; *n* = 10/group. (C–E) Open field test results suggested that the blockade of LXR counteracted the reduction in anxiety‐like behaviors induced by Efavirenz (to some extent) at 28 days after TBI; *n* = 10/group. (F, I) The colocalization of microglia (Iba1, red) with BODIPY (green) and the number of Iba1^+^BODIPY^+^ cells around the lesion at 28 days after TBI; scale bar = 20 μm; *n* = 6/group. (G–J) Colocalization of oligodendrocytes (Olig2, green) with BrdU (red) and the number of Olig2^+^BrdU^+^ cells in the external capsule on the injured side at 28 days after TBI; scale bar = 20 μm; *n* = 6/group. (H–K) Colocalization of OPCs (APC, green) with BrdU (red) and the number of APC^+^BrdU^+^ cells in the external capsule on the injured side at 28 days after TBI; scale bar = 20 μm; *n* = 6/group. *p < 0.05 versus the sham group; *#p* < 0.05 versus the Vehicle group.

Previous research has confirmed that LXR is vital for regulating sterol metabolism in myelin remodeling [5]. To further investigate the underlying mechanism of this effect, GSK2033 (an inhibitor of LXR) was used in this study. Consistent with previous results, CYP46A1 was observed to play a neuroprotective role in foot fault and cylinder experiments, as evidenced by the finding that CYP46A1 activation alleviated foot fault rates and forelimb asymmetry at 28 days after TBI (Figure 6A,B, *p* < 0.05). However, compared with treatment with Efavirenz, treatment with GSK2033 partially attenuated these effects in Efavirenz+GSK2033 mice (Figure 6A,B, *p* < 0.05). Afterward, we conducted an open field test at 28 days after TBI in these three groups. We observed that Efavirenz alleviated anxiety‐like behaviors in TBI model mice, as evidenced by increased exploration time in the center zone (Figure 6C,D, *p* < 0.05), and the inhibition of LXR partially attenuated these effects (Figure 6C,D, *p* < 0.05). However, no differences in total movement time were observed between the groups (Figure 6C–E, *p* > 0.05).

The increase in cholesterol in microglia was subsequently assessed in the TBI model using immunofluorescence. Consistent with earlier findings, fewer BODIPY^+^Iba1^+^ cells were detected in the Vehicle‐treated group than in the Efavirenz‐treated group (Figure 6F–I, *p* < 0.05). However, compared with Efavirenz treatment alone, the blockade of LXR activity with GSK2033 led to a notable increase in the number of BODIPY^+^Iba1^+^ cells in the Efavirenz+GSK2033 group (Figure 6F–I, *p* < 0.05).

To explore the underlying mechanisms of this effect, we assessed the proliferation of oligodendrocytes and OPCs following TBI using BrdU labeling. Aligning with previous findings, the numbers of Olig2^+^BrdU^+^ and NG2^+^BrdU^+^ cells were greater in the Efavirenz‐treated group than in the Vehicle group at 28 days after injury (Figure 6G–K, *p* < 0.05). However, compared with Efavirenz alone, the blockade of LXR signaling with GSK2033 significantly reduced the numbers of Olig2^+^BrdU^+^ and NG2^+^BrdU^+^ cells (Figure 6G–K, *p* < 0.05). These findings indicate that the neuroprotective role of CYP46A1 may involve the LXR signaling pathway, thereby mitigating white matter injury following TBI.

### 3.8 Via the Facilitation of Microglial Clearance of Myelin Debris and the Boosting of Cholesterol Efflux, Efavirenz Establishes a Pro‐Regenerative Milieu That Promotes De Novo Cholesterol Synthesis by Oligodendrocytes and Remyelination

To investigate the mechanisms by which CYP46A1 promotes oligodendrogenesis, proteomic profiling was performed. Gene Ontology (GO) enrichment analysis revealed significant differences in cholesterol‐related processes between the wild‐type (WT) and CYP46A1 knockout (KO) groups, including the modulation of cholesterol transport, efflux, biosynthesis, and homeostasis (Figure S2A). Further examinations revealed that the knockout of CYP46A1 led to reduced expression of multiple enzymes involved in cholesterol biosynthesis, including HMGCS1, HMGCS2, Cyp51a1, Mvd, Mvk, NsdhI, Idi1, Dhcr7, and Fdft1, with HMG‐CoA synthetase (HMGCS1) exhibiting the most pronounced decrease (Figure S2B,C). Additionally, KEGG pathway analysis revealed 12 pathways that were significantly altered upon CYP46A1 deletion, among which the PPAR signaling pathway was notably associated with cholesterol metabolism (Figure S2D); additionally, the results of the PPAR transcription factor assay demonstrated that Efavirenz treatment specifically increased PPARγ activity (but not PPARα or PPARδ activity) (Figure S2E–G).

HMGCS1 plays a crucial role in cholesterol biosynthesis by catalyzing the conversion of acetyl‐CoA into mevalonate. To confirm our proteomics findings, we performed immunofluorescence staining. The results revealed a significant increase in Olig2^+^Hmgcs1^+^ double‐positive cells in the Vehicle group compared with those in the sham group. Furthermore, compared with the Vehicle group, the Efavirenz‐treated group exhibited an even greater number of Olig2^+^Hmgcs1^+^ cells (Figure S2H,I, *p* < 0.05).

PPARγ is a crucial transcription factor that facilitates the differentiation and maturation of OPCs [19, 20]. Studies have demonstrated that rosiglitazone (a PPARγ agonist) induces the production of newly formed mature oligodendrocytes following middle cerebral artery occlusion (MCAO) [21]. Our findings further revealed an increased number of Olig2^+^PPARγ^+^ cells in the Vehicle group compared with that in the sham group, with Efavirenz treatment further increasing this population relative to that in the Vehicle group (Figure S2J,K, *p* < 0.05).

Taken together, these results suggest that CYP46A1 activation stimulates cholesterol biosynthesis in oligodendrocytes via HMGCS1, thereby promoting myelin repair.

## 4 Discussion

In this study, we demonstrated that the activation of CYP46A1 with Efavirenz markedly promotes neurological recovery after TBI and preserves white matter integrity. Mechanistically, Efavirenz increases microglial phagocytic activity and facilitates the clearance of myelin debris through lysosomal degradation. At the molecular level, Efavirenz increases 24OHC levels, which promotes cholesterol efflux through the activation of liver X receptor (LXR) and its downstream targets ABCA1, ApoE, and SREBP2. Consequently, this process reduces cholesterol buildup in microglia, thus creating a regenerative microenvironment that supports oligodendrocyte‐mediated de novo cholesterol synthesis and subsequent remyelination. Importantly, the neuroprotective benefits of Efavirenz were partially diminished upon CYP46A1 knockdown or LXR inhibition, thereby confirming that Efavirenz mitigates white matter damage via its modulation of cholesterol metabolism.

CYP46A1, which is located on chromosome 14q32, encodes the enzyme cholesterol 24‐hydroxylase and is primarily expressed in the brain. This enzyme plays a key role in converting cholesterol into 24OHC, which can cross the blood–brain barrier (BBB) and be metabolized in the liver [22, 23]. Approximately 40%–50% of brain cholesterol turnover is mediated by CYP46A1 [24]. Impaired CYP46A1 function has been linked to various CNS disorders, including Huntington's disease and ischemic stroke [23, 24, 25, 26, 27]. However, its involvement in TBI remains poorly understood.

According to functional tests, CYP46A1 activation via Efavirenz improved neurological outcomes and reduced white matter injury following TBI. Further analysis revealed that this effect was associated with decreased intracellular cholesterol levels, thus suggesting that the CYP46A1/24OHC pathway protects against white matter damage by regulating microglial cholesterol homeostasis. Our findings revealed that Efavirenz treatment upregulated LXR target genes (ABCA1 and ApoE) while downregulating SREBP2, thus reinforcing the role of CYP46A1 in modulating cholesterol balance through LXR and SREBP2 signaling. Although CYP46A1 may influence other pathways [28], our data demonstrate LXR inhibition as a vital regulator of cholesterol metabolism in TBI, thereby indicating the 24OHC/LXR axis as a promising target for TBI treatment.

Analysis of the structure of CYP46A1 indicated that distinct compounds can bind the active site of the enzyme [29]. Efavirenz (a drug that can easily cross the BBB and has been applied in the treatment of AIDS) was demonstrated to bind to the allosteric site of CYP46A1. Efavirenz changes the shape of the active site of cholesterol‐free CYP46A1; as a result, cholesterol binds more tightly to CYP46A1, and enzyme decomposition is more efficient [30]. Moreover, the oral administration of Efavirenz has been observed to enhance long‐term spatial memory, reduce the content of amyloid‐β and prolong the survival of Alzheimer's disease model mice [30, 31]. Recently, Efavirenz has been confirmed as having the ability to decrease phosphorylated Tau (pTau) accumulation in an AD model [32]. Conversely, Efavirenz has been demonstrated to exhibit antitumor effects in prostate carcinoma and ovarian carcinoma [33, 34, 35]. However, the therapeutic efficacy of Efavirenz in TBI remains unclear. Our results demonstrate that Efavirenz plays a neuroprotective role by reducing white matter injury after TBI.

Cholesterol in the brain is primarily produced via de novo synthesis, and this process involves various CNS cell types, due to the fact that the blood–brain barrier blocks the entry of peripheral cholesterol. However, after TBI, the increased permeability of the blood–brain barrier allows cholesterol and cholesterol transport proteins (such as apolipoprotein A1 and ApoE) to enter the brain tissue more readily, leading to the accumulation of cholesterol in the brain. In adults, approximately 70%–80% of brain cholesterol is found in myelin membranes, which are lipid‐rich structures [36]. Myelin consists of multiple layers of oligodendrocyte membranes that encase neuronal axons, thus providing insulation for efficient nerve signal transmission. Mammalian brains exhibit some of the highest cholesterol synthesis rates in the body [36]. Cholesterol availability is a critical factor for myelination. Moreover, oligodendrocytes produce most of the cholesterol required for this process [37].

In conditions such as multiple sclerosis (MS), damage to myelin is triggered by its removal by phagocytes, followed by the regeneration of new myelin sheaths from existing or newly formed oligodendrocytes. Studies in MS models have demonstrated that demyelination and remyelination occur through stages involving phagocyte activation, the clearance of myelin debris, and the creation of a pro‐regenerative environment [38]. Among CNS cells, microglia exhibit the strongest phagocytic activity, whereas astrocytes also participate in myelin clearance. Recent studies have found that astrocytes are also efficient phagocytes of myelin debris. After engulfing the debris, astrocytes can enter a state that is conducive to repair, removing obstacles and “clearing the battlefield” for myelin regeneration [39]. The breakdown and processing of myelin lipids in these cells rely on autophagy mechanisms [40, 41, 42]. Due to the fact that myelin debris can hinder remyelination [43], its lysosomal degradation in phagocytes is essential for repairing damaged areas. Additionally, the recycling and redistribution of internalized lipids to oligodendrocytes may further aid in remyelination.

According to our results, Efavirenz promoted microglial phagocytosis and myelin debris clearance via the lysosomal degradation pathway. CYP46A1 activation also promoted cholesterol efflux via the LXR pathway through 24OHC. Together, these processes lead to the establishment of a local pro‐regenerative microenvironment and promote the de novo synthesis of cholesterol by oligodendrocytes for remyelination.

Previous studies have demonstrated that Efavirenz (at a dosage of 0.09 mg kg‐1 day‐1) activates CYP46A1 in vivo, increasing brain cholesterol turnover [30]. Qiang Zhao et al. have shown that modulating Cyp46a1 by efavirenz intervention (at a dosage of 0.09 mg kg‐1 day‐1) reduces chronic neuroinflammation, highlighting a potential therapeutic approach for promoting recovery after ischaemic stroke in mice. Collectively, these findings indicate that efavirenz treatment at a low dosage is effective and safe [44]. Therefore, in this study, we choose a dosage of 0.1 mg kg‐1 day‐1 as a therapeutic dose.

Efavirenz is an anti‐HIV medication (belonging to non‐nucleoside reverse transcriptase inhibitors (NNRTIs)) known to trigger CYP46A1 activity by binding to the enzyme's allosteric site [30]. Bioinformatic chemical–protein interaction network analysis (STITCH) indicated a strong association between Efavirenz and the CYP46A1 protein [30]. Therefore, Efavirenz exerts its neuroprotective effect by activating CYP46A1.

Due to factors such as its mechanism of action, clinical evidence, and safety profile, Efavirenz currently lacks a reasonable basis for its direct repurposing towards the treatment of TBI. If its potential role in TBI is to be explored, rigorous clinical trials are required for validation.

Efavirenz has been observed to be associated with adverse neuropsychiatric events (NPAEs) at high doses [45]. However, such side effects were not reported at lower doses, thus indicating a potential therapeutic window for safe use [35].

This study has several limitations. First, only male mice were used, so the conclusions may not be generalizable to female populations. Second, the lack of cell‐specific manipulation of CYP46A1 may obscure cell‐type‐specific mechanisms. Third, the limited temporal resolution of the experiments precluded clarification of the temporal relationship between cholesterol metabolism and remyelination. Lastly, the study did not directly test the causal link between microglial lipid metabolism and remyelination; specifically, the direct causal relationship between microglial cholesterol efflux and oligodendrocyte remyelination was not sufficiently demonstrated, and the underlying cell‐type‐specific mechanisms remain unclear.

In conclusion, our findings demonstrate that CYP46A1 plays a critical role in regulating cholesterol homeostasis in microglia and that the targeting of CYP46A1 may offer a viable therapeutic approach for TBI. The CYP46A1 activator Efavirenz reduces white matter damage by increasing the levels of the cholesterol metabolite 24OHC, thereby stabilizing the cholesterol balance after TBI. Furthermore, Efavirenz (a widely used antiretroviral drug) could serve as a promising treatment for TBI by restoring 24OHC/LXR signaling.

## Author Contributions

Lin Li, Yujie Chen, and Zhi Chen conceived and designed the study. Lin Li, You Shi, Qing Luo, Peiwen Guo, Taotao Jin, Zhouyang Jiang, Yin Niu, and Wenyan Li acquired and analyzed the data. Lin Li, You Shi, Qing Luo, and Yujie Chen drafted a substantial portion of the manuscript. All authors, including Xufang Ru, read and approved the final version of the manuscript for publication.

## Funding

This work was supported by the National Natural Science Foundation of China (Grant No. 82371361 to Zhi Chen, 82371333 to Yujie Chen), the Natural Science Foundation of Chongqing (Grant No. CSTB2025NSCQ‐LZX0044 to Yujie Chen), the Chongqing Municipal Health Commission (Grant No. YXGD202451 to Yujie Chen), and the Chongqing Science & Technology Bureau (Grant No. cstc2024ycjh‐bgzxm0103 to Peiwen Guo). The funders of the study had no role in study design, data collection, data analysis, data interpretation, or the writing of the report.

## Ethics Statement

All the animal experiments were reviewed and approved by the Laboratory Animal Welfare and Ethics Committee of the Army Medical University (AMUWEC20224973); moreover, the experiments were performed according to the Guidelines for the Care and Use of Laboratory Animals of the National Institutes of Health, and results were reported in accordance with the ARRIVE 2.0 guidelines.

## Conflicts of Interest

The authors declare no conflicts of interest.

## Supporting information


**Figure S1:** CYP46A1 activation restored the structural integrity of white matter after TBI.
**Figure S2:** Efavirenz promoted microglial clearance of myelin debris and cholesterol efflux, thereby establishing a pro‐regenerative environment that stimulates oligodendrocytes to produce cholesterol and supporting remyelination.

## Data Availability

The data that support the findings of this study are available from the corresponding author upon reasonable request.
