# Supplementary material for: CYP46A1‐Targeted Treatment Alleviates Long‐Term White Matter Injury Following Traumatic Brain Injury by Promoting Cholesterol Metabolic Clearance and Remyelination
Source: CNS Neurosci Ther. 2026 Jun 15;32(6):e70984. doi: 10.1002/cns.70984 (PMC13269851; doi:10.1002/cns.70984)
Supplement: Supplementary file 1 — Figure S1: CYP46A1 activation restored the structural integrity of white matter after TBI. Figure S2: Efavirenz promoted microglial clearance of myelin debris and cholesterol efflux, thereby establishing a pro‐regenerative environment that stimulates oligodendrocytes to produce cholesterol and supporting remyelination. [file CNS-32-e70984-s001.docx]

**CYP46A1-targeted treatment alleviates long-term white matter injury following traumatic brain injury by promoting cholesterol metabolic clearance and remyelination**

Runing Title: CYP46A1-targeted treatment alleviates TBI

Lin Li^1, 4, 5, #^, You Shi^2, #^, Qing Luo^3, #^, Peiwen Guo^1, 4, 5^, Taotao Jin^1, 4, 5^,
Xufang Ru^1, 4, 5^, Zhouyang Jiang^1, 4, 5^, Yin Niu^1, 4, 5^, Wenyan Li^1, 4, 5^,
Yujie Chen^1, 4, 5, *^, Zhi Chen^1, 4, 5, *^

1. *﻿*Department of Neurosurgery, Southwest Hospital, Third Military Medical University (Army Medical University), Chongqing, 400038, China.
2. Department of Neuro-oncology, Chongqing University Cancer Hospital, Chongqing, 400030, China.
3. Department of Ultrasound，Chonggang General Hospital, Chongqing University of Posts and Telecommunications, Chongqing, 400080, China.
4. Chongqing Key Laboratory of Intelligent Diagnosis, Treatment and Rehabilitation of Central Nervous System Injuries, Southwest Hospital, Third Military Medical University (Army Medical University), Chongqing, 400038, China.
5. Chongqing Clinical Research Center for Neurosurgery, Southwest Hospital, Third Military Medical University (Army Medical University), Chongqing, 400038, China.

^#^These authors contributed equally to this work.

*Correspondence to:

**Yujie Chen, MD, PhD**

E-mail: chenyj@tmmu.edu.cn; [yujiechen6886@foxmail.com](mailto:yujiechen6886@foxmail.com)

ORCID: 0000-0002-9905-9138

Phone: (86)023-68765925, Fax: (86)023-68765922

**Zhi Chen, MD, PhD**

E-mail: zhichen@tmmu.edu.cn

ORCID: 0000-0002-8404-4937

Phone: (86)023-68765902, Fax: (86)023-68765922

Department of Neurosurgery, Southwest Hospital, Third Military Medical University (Army Medical University). 29 Gaotanyan Street, Shapingba District, Chongqing, 400038, China.

**Supplemental Methods**

**Controlled cortical impact (CCI) model induction and grouping**

A controlled cortical impact (CCI) device (Precision Systems and Instrumentation, TBI-0310, USA) was used to induce TBI^1^. Following anesthesia, a 3 mm circular craniotomy was performed on the right parietal cortex, positioned 1 mm posterior to the bregma and 1 mm lateral to the sagittal suture. The CCI injury was then administered using the following standardized parameters: impact velocity (5.0 m/s), penetration depth (2.0 mm), and dwell time (100 ms). This procedure produced a moderately severe contusion in the right sensorimotor cortex. The sham-operated group underwent the same surgical steps but without the impact. After the procedure, the craniotomy site was sealed with bone wax, and the incision was sutured. Finally, the animals were placed on a heating pad to maintain a normal temperature until recovery.

The mice were randomly allocated to three experimental groups: the sham group (n = 40), the TBI + vehicle group (n = 40), and the TBI + Efavirenz group (n = 40). The sham group received only a craniotomy, while the vehicle and efavirenz (a CYP46A1 activator) groups were subjected to CCI. The vehicle group was administered an equivalent volume of the control solution, whereas the Efavirenz group received Efavirenz (Stocrin, Merck Sharp & Dohme, Australia) starting 1 hour post-TBI (orally administered at 0.1 mg/kg/day for a period of 14 days)^2^.

To investigate whether CYP46A1 depletion affects the neuroprotective effects of efavirenz after TBI, 60 CYP46A1 knockout (KO) mice were divided into the following groups: the KO + Sham (n = 20 group), KO + Vehicle (n = 20 group), and KO + Efavirenz (n = 20 group) groups.

To investigate the underlying mechanism, the LXR inhibitor GSK2033 (Sigma‒Aldrich, #D2650) was administered via intracerebroventricular infusion (0.3 mg/kg, dissolved in 5 μL of 1% DMSO) 30 min prior to TBI^3^, and the mice were randomly assigned to three groups: the TBI + vehicle (vehicle) group (n = 16), the TBI+ efavirenz (efavirenz) group (n = 16) and the TBI+ efavirenz+ GSK2033 (efavirenz+ GSK2033) group (n=16).

**Foot fault test**

To estimate sensorimotor coordination during spontaneous movement, a foot fault experiment was carried out as previously detailed^4^. The mice walked for one minute on an elevated grid (dimensions: 40 cm length × 20 cm width × 30 cm height) featuring 1.5 cm × 1.5 cm square openings (area: 2.25 cm²). The session was recorded from beneath the grid. A blinded observer subsequently analyzed the recordings to determine both the total number of steps taken (indicating general locomotion) and the number of foot faults specifically involving the left forepaw (the impaired limb). A foot fault was defined as an instance where the left forepaw was misplaced and slipped through an opening. Fault frequency was expressed as a percentage of total steps.

﻿**Cylinder test**

In this experiment, postoperative forelimb asymmetry^4^ was assessed. The mice were placed in a clear-walled cylinder (9 cm diameter, 15 cm height), and their behavior was video recorded from above for 8 to 10 minutes. During spontaneous exploration, mice typically reared and contacted the cylinder wall using their right forelimb (R), left forelimb (L), or both forelimbs simultaneously (B). An investigator who was blinded to the treatment groups recorded and analyzed the behavior. Given the right-hemisphere brain injury, the preference for the left forelimb was calculated as (L + B/2)/(L + R + B) × 100%. Animals demonstrating fewer than 10 rears per session or a baseline preference for either forelimb prior to TBI were excluded from subsequent analysis.

**Morris water maze test**

Cognitive function was evaluated using the Morris water maze test^5^. In the learning phase, the mice navigated an opaque water-filled circular pool (109 cm diameter) to find a hidden square platform (11 × 11 cm²) submerged 2 cm above the water surface. Each day for five consecutive days, the mice underwent three training trials with randomized starting positions, allowing 60 seconds per trial to locate the platform. After each trial, the mice remained on the platform for 30 seconds with salient spatial cues visible around the room. Escape latency (time to reach the platform) was recorded on day 28 post-TBI. For the memory probe test, the platform was removed, and the mice swam freely for 60 seconds. The time spent in the target quadrant where the platform had been located was recorded.

**Open field test**

The experiment was carried out in an open field arena (a 100 cm × 100 cm × 40 cm enclosure). On day 28, the animals were positioned at the center of the apparatus, and their behavior was monitored for 5 minutes via a video-tracking system (ANY-maze, USA). The total duration of movement and the time spent in the central area of the arena were assessed by an experimenter unaware of the treatment groups^5^.

**Electrophysiologic recording**

To measure motor evoked potentials (MEPs) on day 28, the animals were anesthetized using 1% sodium pentobarbital (25 mg/kg, intraperitoneal injection). A needle stimulation electrode (Medtronic, DSN1620, USA) was placed beneath the skin near the nasal bridge, with its tip contacting the scalp surface as the positive pole. The negative pole was positioned at the center point between both ears. A reference electrode was implanted under the skin near the tail region. For signal acquisition, a recording electrode was placed in the left (impaired limb) gastrocnemius muscle. Brain excitation was achieved using an electrical stimulator (Medtronic, Keypoint, USA), which delivered repeated pulse stimuli (10 mA intensity, 0.1 ms duration, 1 Hz frequency). Each animal received a minimum of five stimulations with 15-second intervals between applications. The MEP value was determined by measuring the vertical amplitude from baseline to peak for individual stimulus responses^5^.

**Transmission electron microscopy**

Transmission electron microscopy (TEM) was employed to evaluate myelin thickness in the corpus callosum/external capsule (CC/EC) surrounding the injury site. First, the mice were transcardially perfused with chilled saline, followed by a fixative solution containing 4% paraformaldehyde (PFA) and 2.5% glutaraldehyde in 0.1 M PBS. Subsequently, CC/EC tissues adjacent to the injured region were carefully dissected into small blocks (~1 mm³). These tissue samples were then postfixed by immersion in 2% glutaraldehyde for 24 hours. After the samples were rinsed with PBS, they were treated with 1% osmium tetroxide in 0.1 M PBS for 45 minutes. Dehydration was carried out using a graded acetone series, followed by embedding in Araldite resin. Ultrathin sections (60 nm) were prepared using a Leica UCT ultramicrotome equipped with a diamond knife (Diatome, Germany). The sections were subsequently stained with uranyl acetate and lead citrate before being examined under a JEM-1400Plus transmission electron microscope at 20,000× magnification. For g-ratio analysis, randomly selected CC/EC regions were imaged, and the ratio of the inner axonal diameter to the total outer diameter (axon + myelin) was determined using ImageJ software^5^.

**Magnetic resonance imaging**

To evaluate white matter injury at 28 days post-TBI^6^, diffusion tensor imaging (DTI) was performed. The mice received anesthesia induction with 3% isoflurane and were secured in a stereotaxic head holder. Throughout the scanning procedure, respiration and body temperature were continuously monitored, while anesthesia was maintained using 1%-1.5% isoflurane. The DTI acquisition parameters were as follows: TE = 0.85 ms, TR = 5000 ms, matrix size = 256 × 128, slice thickness = 0.8 mm, 32 diffusion directions, and b value = 826.59 s/mm.

Data analysis was performed with ParaVision 5.0 software (Bruker BioSpin, Germany). Regions of interest (ROIs) encompassing the corpus callosum and external capsule (CC/EC) on the injured hemisphere were manually outlined. Fractional anisotropy (FA) values were computed using the formulas FA=$\sqrt{\frac{3}{2}\frac{\left( \lambda1-MD \right)^{2}+\left( \lambda2-MD \right)^{2}+\left( \lambda3-MD \right)^{2}}{{\lambda1}^{2}+{\lambda2}^{2}+{\lambda3}^{2}}}$ and $MD=\frac{\lambda1+\lambda2+\lambda3}{3}$.

Directionally encoded color (DEC) maps and FA maps were generated using ParaVision software.

**Microglial magnetic sorting**

Microglial magnetic sorting was carried out on day 28 post-TBI. First, the animals were transcardially perfused with ice-cold PBS. Afterward, the brains were harvested and rinsed with Hank’s balanced salt solution (HBSS; Gibco, USA). After the meninges were removed, a Neural Tissue Dissociation Kit P (Miltenyi Biotec, Germany) was used for enzymatic cell dissociation according to the manufacturer's guidelines. After being digested by papain, the tissues were homogenized, dissociated, and passed through a 40-µm cell strainer. To remove myelin, the suspension was subsequently centrifuged at 850 g for 10 minutes at 4°C in 10 mL of cold 0.9 M sucrose solution, after which the supernatant and floating myelin were discarded. The cell pellet was prepared for microglial magnetic sorting by incubation with CD11b MicroBeads (1:10 in PBS + 0.05% BSA; Miltenyi Biotec, Germany) for 15 minutes at 4°C. After being washed with PBS, the cells were resuspended in 500 µL of PBS + 0.05% BSA and passed through a magnetic column to purify the CD11b+ cells. Cell counts were conducted via a TC20 Automated Cell Counter (Bio-Rad, USA) according to the manufacturer's instructions ^7^. The purity of the isolated microglia was evaluated using flow cytometry^8^. Four samples of CD11b+ cells were processed by immunomagnetic beads; one sample was treated with FITC-labeled anti-CD11B and APC-labeled anti-CD45 antibodies, while another sample served as a negative control without antibodies. The remaining samples served as positive controls. After 30 minutes of incubation in the dark, the cells were washed, after which the purity of the CD11b+CD45+ cells was evaluated via flow cytometry^6^.

**Immunofluorescence staining (IF) and BODIPY staining**

Brain tissue samples from mice were processed for immunofluorescence staining at 28 days after TBI induction^9^. The animals were euthanized and subjected to intracardiac perfusion with PBS, followed by fixation with 4% paraformaldehyde (PFA). After 24 hours of postfixation in PFA, the tissues were cryoprotected by immersion in a 30% sucrose solution at 4°C for 3–5 days. Coronal brain sections (20 μm thick) were obtained and rinsed with PBS. Permeabilization was carried out using 0.4% Triton X-100 for 30 min, followed by blocking with 10% goat serum at 37°C for 1 h. The sections were then incubated with primary antibodies overnight at 4°C. After being washed with PBS, they were treated with appropriate secondary antibodies for 1 h at 37°C. For the double-labeling experiments, the same steps were repeated with another primary antibody. The following primary antibodies were used: goat anti-Iba1 (Abcam #ab5076, 1:200), rabbit anti-CD68 (Abcam, #ab125212, 1:200), rat anti-LAMP1 (Abcam, #ab25245, 1:200), chicken anti-MBP (Sigma, #MAB9348, 1:200), rabbit anti-MBP (Sigma‒Aldrich, #AB5864, 1:200), mouse anti-neurofilament H (Sigma‒Aldrich, #N5389, 1:100), rabbit anti-Nav1.6 (Abcam, #ab65166, 1:200), rabbit anti-NG2 (Biorbyt, #orb382135, 1:100), mouse anti-APC (Sigma‒Aldrich, #OP80, 1:100), and mouse anti-Caspr (Sigma‒Aldrich, #MABN69, 1:300).

Fluorescence images were acquired using a Zeiss LSM780 confocal microscope, with at least six images per animal. Quantitative analysis of the immunopositive cells was conducted using ImageJ software.

The nodes of Ranvier (NOR) in the external capsule (EC) or corpus callosum (CC) were accessed on day 28 post-TBI by immunostaining for Caspr1 and Nav1.6, which identified the paranodal and nodal regions, respectively^10^. All counts were performed in a blinded manner.

For BODIPY staining, samples were rinsed once with PBS (5 min, 37°C) and then treated with BODIPY™ 493/503 (Thermo Fisher Scientific, D2191, diluted 1:1000 from a 1 mg/mL DMSO stock) in PBS to label lipid droplets.

**TUNEL staining**

To evaluate cell death, a TUNEL assay (Thermo Fisher Scientific, C10625) was conducted. First, the samples were rinsed and permeabilized. A TUNEL reaction mixture was prepared by combining 50 μL of enzyme solution with 450 μL of label solution, yielding a total volume of 500 μL. The samples were then incubated with this mixture for 60 minutes at 37°C under light-protected conditions. Finally, fluorescent double staining was performed ^5^.

**BrdU injections**

To mark proliferating cells ﻿beginning on the third day post-surgery, the mice were intraperitoneally injected with ﻿bromodeoxyuridine (BrdU) (Sigma‒Aldrich, MSM1X-469-P1ABX, 50 mg/kg) twice a day with a ﻿minimum interval of 8 h for 5 consecutive days ^4^.

**Western blot analysis**

Western blotting was performed on day 28 after surgery. Tissue samples from the pericontusional area were homogenized in RIPA buffer supplemented with protease and phosphatase inhibitors and lysed for 1 hour at 4°C. After centrifugation (12,000 × g, 15 min, 4°C), the supernatant was collected. Protein samples (50 µg per lane) were separated by SDS‒PAGE and then electrotransferred onto PVDF membranes. The membranes were blocked with 5% nonfat milk for 1 h at room temperature before they were incubated with primary antibodies at 4°C overnight. The primary antibodies used were as follows: rabbit anti-LXR (Abcam, #ab315082, 1:1000), rabbit anti-Abca1 (CST, #96292, 1:1000), rabbit anti-ApoE (CST, #49285, 1:1000), rabbit anti-SREBP2 (NOVUS, #NB100-74543, 1:1000), and mouse anti-β-actin (Proteintech, #66009-1-Ig, 1:1000). After being washed, the membranes were incubated with appropriate HRP-conjugated secondary antibodies. Protein bands were detected using a Fusion-FX7 imaging system (Vilber Lourmat, China) and quantified with ImageJ software ^9^.

**HPLC‒MS analysis and cholesterol measurements**

﻿ The serum concentration of 24S-OHC was measured on day 28 after TBI using HPLC‒MS following established methods^11^. Briefly, 50 μl aliquots of serum or brain homogenate were processed through acid hydrolysis and derivatization steps, followed by solvent evaporation using a vacuum drying system. For HPLC‒MS analysis, the dried samples were reconstituted in 1 ml of methanol–water (9:1, v/v) solution and then centrifuged at 2,400 × g for 15 minutes (4°C). The resulting supernatant was transferred to glass vials for subsequent analysis.

To determine the intracellular cholesterol levels, an adapted version of the Amplex Red Cholesterol Assay Kit protocol (Thermo Fisher Scientific, A12216) was used as described in the manufacturer's guidelines ^12^.

**Proteomic analysis**

At 28 days post-TBI, tissue samples adjacent to the injured area were collected and subsequently submitted to PTM BIO Co. (Hangzhou, China) for proteomic profiling. Protein extraction and quantification were performed first, followed by separation and enzymatic digestion. The processed proteins were then subjected to analysis using a nanoElute system interfaced with a timsTOF Pro mass spectrometer (Bruker, Germany). For MS data processing, MaxQuant (version 1.6.15.0) was used, with statistically significant thresholds set at a fold change of >2 or <0.5 and a p value <0.05^13^.

**PPAR activity assay**

Nuclear extracts were isolated from the tissue surrounding the lesion utilizing the Nuclei PURE Prep Extraction Reagent Kit (Merck, NUC201). Peroxisome proliferator-activated receptor (PPAR (α, δ, γ)) transcriptional activity was measured with a PPAR Transcription Factor Assay Kit (Abcam, ab133113) in accordance with the manufacturer's protocols^14^.

**References**

1. Zhang Y, Wang L, Pan Q, et al. Selective sphingosine-1-phosphate receptor 1 modulator attenuates blood-brain barrier disruption following traumatic brain injury by inhibiting vesicular transcytosis. *Fluids Barriers CNS*. Jul 11 2022;19(1):57. doi:10.1186/s12987-022-00356-6

2. Han M, Wang S, Yang N, et al. Therapeutic implications of altered cholesterol homeostasis mediated by loss of CYP46A1 in human glioblastoma. *EMBO Mol Med*. Jan 9 2020;12(1):e10924. doi:10.15252/emmm.201910924

3. Chen L, Song D, Chen B, Yang X, Cheng O. Activation of liver X receptor promotes hippocampal neurogenesis and improves long-term cognitive function recovery in acute cerebral ischemia-reperfusion mice. *J Neurochem*. Jul 2020;154(2):205–217. doi:10.1111/jnc.14890

4. Pu H, Zheng X, Jiang X, et al. Interleukin-4 improves white matter integrity and functional recovery after murine traumatic brain injury via oligodendroglial PPARgamma. *J Cereb Blood Flow Metab*. Mar 2021;41(3):511–529. doi:10.1177/0271678X20941393

5. Li L, Luo Q, Shang B, et al. Selective activation of cannabinoid receptor-2 reduces white matter injury via PERK signaling in a rat model of traumatic brain injury. *Exp Neurol*. Jan 2022;347:113899. doi:10.1016/j.expneurol.2021.113899

6. Zhang Y, Shi Y, Wang L, et al. TREM2 activation reduces white matter injury via PI3K/Akt/GSK-3beta signalling after intracerebral haemorrhage. *Br J Pharmacol*. Jun 2025;182(11):2542–2559. doi:10.1111/bph.17475

7. Villa A, Gelosa P, Castiglioni L, et al. Sex-Specific Features of Microglia from Adult Mice. *Cell Rep*. Jun 19 2018;23(12):3501–3511. doi:10.1016/j.celrep.2018.05.048

8. Yang H, Ni W, Wei P, et al. HDAC inhibition reduces white matter injury after intracerebral hemorrhage. *J Cereb Blood Flow Metab*. May 2021;41(5):958–974. doi:10.1177/0271678X20942613

9. Yan J, Zhang Y, Wang L, et al. TREM2 activation alleviates neural damage via Akt/CREB/BDNF signalling after traumatic brain injury in mice. *J Neuroinflammation*. Dec 3 2022;19(1):289. doi:10.1186/s12974-022-02651-3

10. Zhang W, Pu H, Hu X, et al. Poststroke Intravenous Transplantation of Human Mesenchymal Stem Cells Improves Brain Repair Dynamics and Functional Outcomes in Aged Mice. *Stroke*. Apr 2023;54(4):1088–1098. doi:10.1161/STROKEAHA.122.041507

11. Wang T, Zhang X, Wang Y, et al. High cholesterol and 27-hydroxycholesterol contribute to phosphorylation of tau protein by impairing autophagy causing learning and memory impairment in C57BL/6J mice. *J Nutr Biochem*. Aug 2022;106:109016. doi:10.1016/j.jnutbio.2022.109016

12. Wiener JP, Desire S, Garliyev V, Lyssenko Iii N, Pratico D, Lyssenko NN. Down-Regulation of ABCA7 in Human Microglia, Astrocyte and THP-1 Cell Lines by Cholesterol Depletion, IL-1beta and TNFalpha, or PMA. *Cells*. Aug 25 2023;12(17)doi:10.3390/cells12172143

13. Li LM, Kodosaki E, Heslegrave A, et al. High-dimensional proteomic analysis for pathophysiological classification of traumatic brain injury. *Brain*. Mar 6 2025;148(3):1015–1030. doi:10.1093/brain/awae305

14. Zhang Q, Zhu W, Xu F, et al. The interleukin-4/PPARgamma signaling axis promotes oligodendrocyte differentiation and remyelination after brain injury. *PLoS Biol*. Jun 2019;17(6):e3000330. doi:10.1371/journal.pbio.3000330


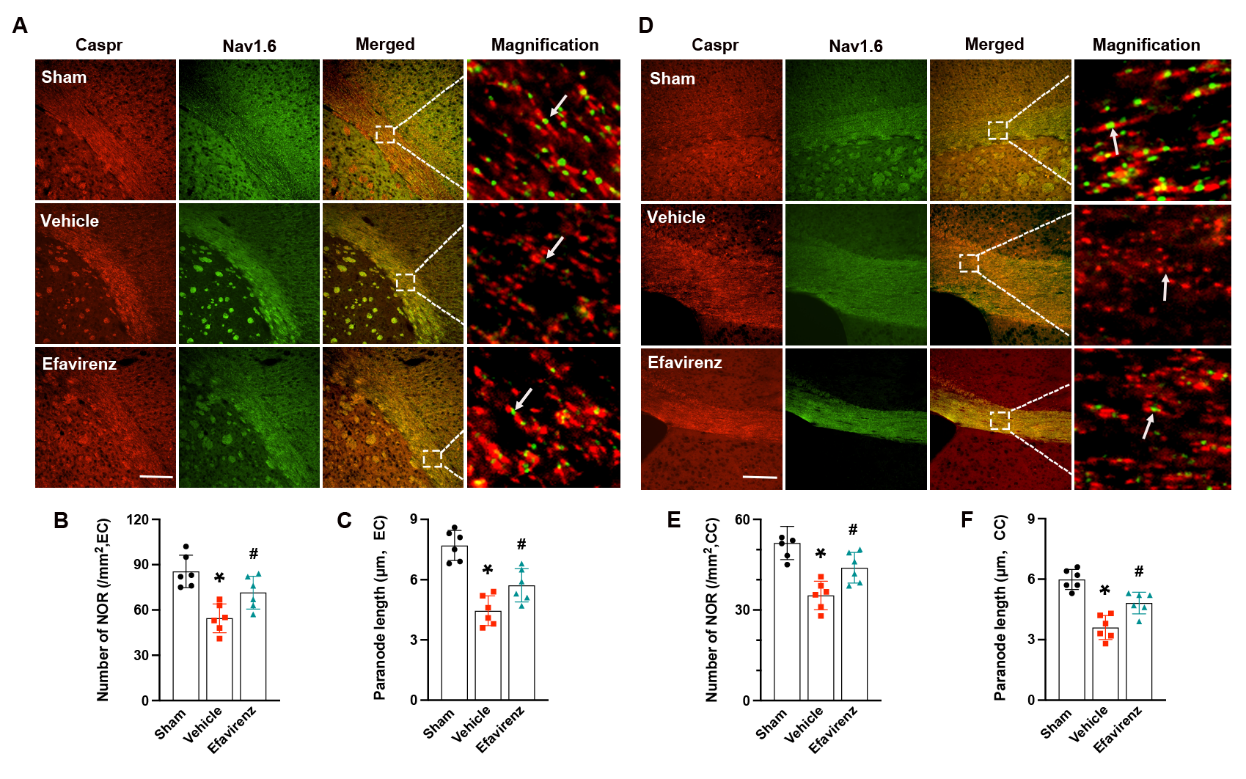


**Supplemental Figure 1. CYP46A1 activation restored the structural integrity of white matter after TBI.**

Immunostaining was performed to evaluate Caspr and Nav1.6 expression in the EC and CC areas 28 days after TBI. (A, D) Images showing Caspr (red) and Nav1.6 (green); scale bar = 20 µm; n=6/group. (B, E) The number of nodes of Ranvier (NOR) was quantified; n=6/group. (C, F) The length of the paranode (Caspr+) was measured; n=6/group. **P < 0.05* ﻿versus the sham group; *#P < 0.05* ﻿versus the vehicle group.


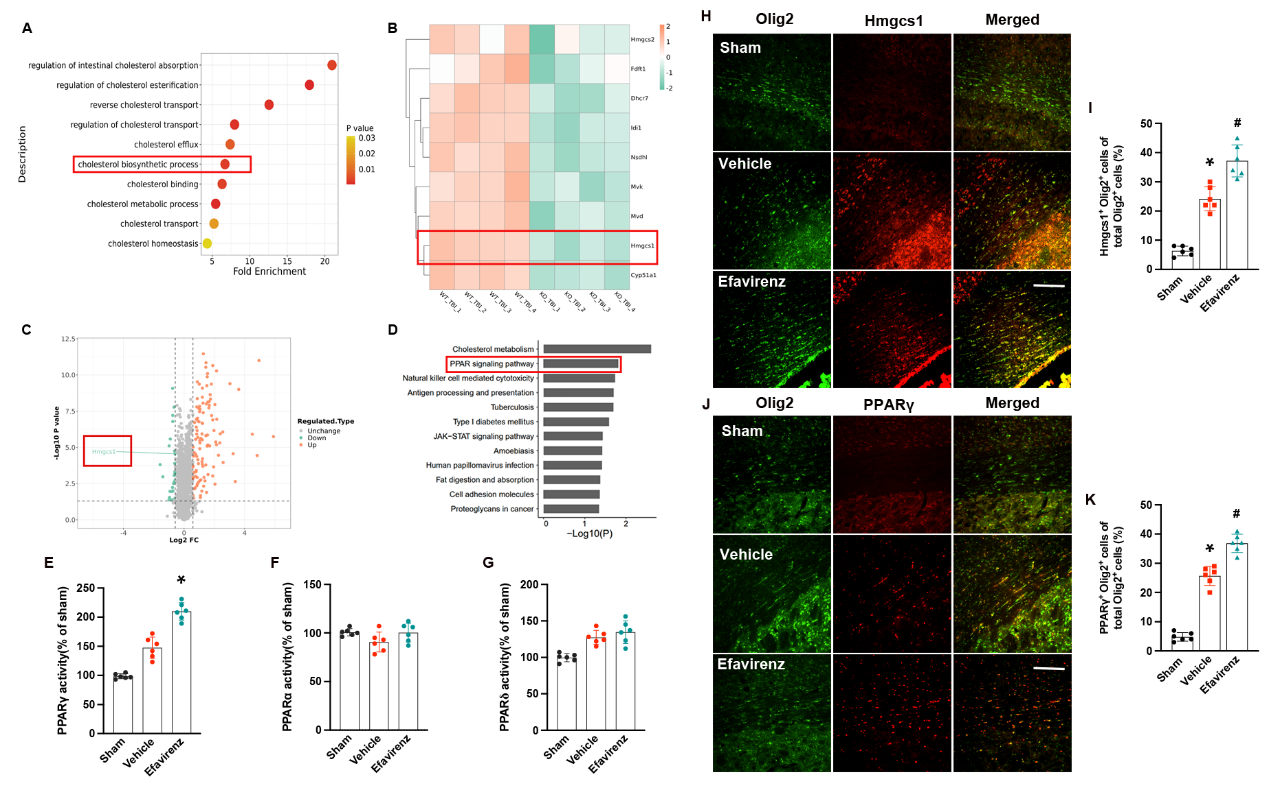


**Supplemental Figure 2. Efavirenz promoted microglial clearance of myelin debris and cholesterol efflux, establishing a proregenerative environment that stimulates oligodendrocytes to produce cholesterol and supporting remyelination.**

(A–D) Proteomic analysis was carried out 28 days after TBI; n=4/group. ﻿(E-G) Measurement of PPAR DNA binding activity; n=6/group. (H, I) Colocalization of oligodendrocytes (Olig2, green) with Hmgcs1 (red) and the number of Olig2^+^ Hmgcs1 ^+^ cells in the external capsule on the injured side at 28 days after TBI; scale bar = 20 µm; n=6/group. (J, K) Colocalization of oligodendrocytes (Olig2, green) with PPARγ (red) and the number of Olig2^+^ PPARγ ^+^ cells in the external capsule on the injured side at 28 days after TBI; scale bar = 20 µm; n=6/group. **P < 0.05* ﻿versus the sham group; *#P < 0.05* ﻿versus the vehicle group.
